# “Amniotic fluid” in womb-like flower bracts protects floral development and promotes drought resistance in karst habitats

**DOI:** 10.1093/nsr/nwaf195

**Published:** 2025-05-16

**Authors:** Yongpeng Ma, Gang Yao, Yongquan Ren, Detuan Liu, Yuanting Shen, Wei Huang, Yuewen Xu, Spencer C H Barrett, Hang Sun, Bo Song

**Affiliations:** Yunnan Key Laboratory for Integrative Conservation of Plant Species with Extremely Small Populations/State Key Laboratory of Plant Diversity and Specialty Crops, Kunming Institute of Botany, Chinese Academy of Sciences, China; Yunnan Key Laboratory for Integrative Conservation of Plant Species with Extremely Small Populations/State Key Laboratory of Plant Diversity and Specialty Crops, Kunming Institute of Botany, Chinese Academy of Sciences, China; College of School of Eco-Environmental Engineering, Guizhou Minzu University, China; Yunnan Key Laboratory for Integrative Conservation of Plant Species with Extremely Small Populations/State Key Laboratory of Plant Diversity and Specialty Crops, Kunming Institute of Botany, Chinese Academy of Sciences, China; Yunnan Key Laboratory for Integrative Conservation of Plant Species with Extremely Small Populations/State Key Laboratory of Plant Diversity and Specialty Crops, Kunming Institute of Botany, Chinese Academy of Sciences, China; Department of Ecology and Evolutionary Biology, University of Toronto, Canada; Yunnan Key Laboratory for Integrative Conservation of Plant Species with Extremely Small Populations/State Key Laboratory of Plant Diversity and Specialty Crops, Kunming Institute of Botany, Chinese Academy of Sciences, China; Department of Ecology and Evolutionary Biology, University of Toronto, Canada; Key Laboratory of Phytochemistry and Natural Medicines, Kunming Institute of Botany, Chinese Academy of Sciences, China; Yunnan Key Laboratory for Integrative Conservation of Plant Species with Extremely Small Populations/State Key Laboratory of Plant Diversity and Specialty Crops, Kunming Institute of Botany, Chinese Academy of Sciences, China

**Keywords:** cooling, drought resistance, flower development, Gesneriaceae, karst

## Abstract

Water serves diverse functions in plant growth and reproduction. Here, we report a novel strategy in the terrestrial karst plant *Hemiboea magnibracteata* analogous to amniotic fluid in mammals. Developing flower buds enclosed in ‘womb-like’ bracts are completely immersed in fluids and protected against excessive temperature and desiccation. The reservoir of water also likely limits drought and calcium stress in the highly specialized abiotic conditions of karst habitats.

The early developmental stages of most organisms (e.g. embryos or flower buds) are more sensitive to adverse influences of the environment than at other times in the life cycle [1,2]. Therefore, the mechanisms by which organisms protect their offspring are an important biological question. Perhaps the most well-known example is the presence of amniotic fluid throughout gestation in mammals. The fluid enables normal development of the fetal respiratory, gastrointestinal and urinary tracts and musculoskeletal system, and enables fetal growth in a nonrestricted, sterile and thermally controlled environment [3]. Although amniotic fluid primarily consists of water, its emergence as an adaptive mechanism protecting developing embryos is a key innovation in vertebrate evolution [4].

Similarly, water serves a variety of functions in plant reproduction during floral development and pollination [5], although water can be detrimental to some reproductive functions, including reducing pollen viability and nectar quality [6,7]. Beneficial functions of water include driving growth and the expansion of flowers, and regulating temperature by transpiration in arid environments [5]. In these circumstances, water mainly exists within the tissues of flowers, although some species produce bowl- or cup-shaped structures that surround flowers and contain significant volumes of rainwater or mucilaginous mixtures, thereby acting as a moat to prevent herbivore access to floral structures [8,9].

We are unaware of reports of terrestrial plants that completely immerse immature flowers in water or other fluids in a manner that is analogous to the amniotic fluid in most vertebrate animals.

*Hemiboea magnibracteata* (Gesneriaceae) is a perennial herb of montane forests that grows on limestone rocks and is endemic to the karst regions of northwestern Guangxi and southern Guizhou province, southwestern China (Fig. 1A and B). Plants flower from July to August and are mainly pollinated by pollen-collecting solitary bees, with each individual producing several flowering stems in a season. Our field observations of populations of *Hemiboea* unexpectedly revealed that each flower stem produces one or two global- or oval-like structures, each formed by fusion of the two opposite bracts, with a developing flower completely immersed in liquid contained in the globe- or oval-like structures (Fig. 1C and D). During the growth of the bracts, continuous fluid secretion was observed at the bract–pedicel junction, lasting for ∼4–5 weeks, with secretory volumes peaking at 17.7 ± 5.9 mL (mean ± SD, *n* = 20) in each globe. The fluids originate primarily from root-absorbed water, undergoing transport through the stem vascular system and ultimately accumulating in the bracts. Flowers subsequently emerge from the bracts owing to dehiscence of the bracts and loss of liquid (Fig. 1E and F). Then, another two globe- or oval-like structures develop in each dehisced globe- or oval-like structure

**Figure 1.**
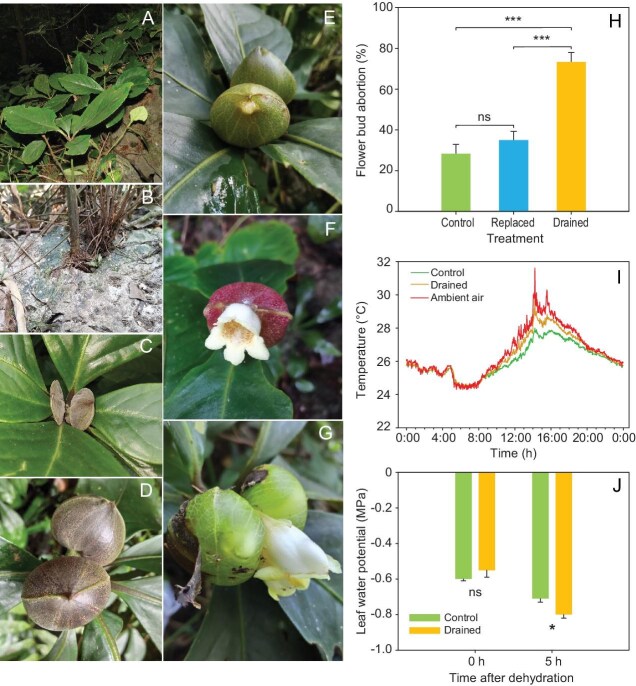
(A, B) Habitat and (C–G) floral bracts of *Hemiboea magnibracteata* and the effects of fluids contained in the closed bracts on (H) flower development, (I) flower temperature and (J) leaf water potential. (B) Typical microenvironment with plants growing out of limestone rock, which is vulnerable to drought stress. (C) Early stage of oval-like bracts. (D) Maximum stage of oval-like bracts. (E) Dehiscent bracts. (F) Protruding flower. (G) Flower and either side of two oval bracts filled with fluids. (H) Percentage of aborted flower buds among unmanipulated control bracts, bracts with fluids drained and bracts with fluids replaced by purified water. (I) Variation in daily temperature for flower buds immersed in fluid (control), for buds in bracts drained of fluid and for ambient air close to flower bracts. (J) Variation in leaf water potential after dehydration between leaves with fluids in neighboring bracts drained and leaves with fluids in neighboring bracts intact. Data are means ± SE. *** and * indicate significant difference at *P* < 0.001 and *P* < 0.05, respectively.

(Fig. 1G). Thus, each flower stem can produce three to six flowers. To the best of our knowledge, this is the first report of flower buds enclosed in ‘womb-like’ structures filled with fluids prior to flowering in a terrestrial plant.

Here, using field observations, manipulative experiments and chemical analyses, we explore the possible adaptive basis of the womb-like structures in *Hemiboea.* Specifically, we investigated several non-mutually exclusive ecological hypotheses that might explain the function of the fluid-filled bracts. These included the role of temperature moderation, herbivore deterrence and both calcium and water uptake. We conducted our investigations from June to October during five consecutive years (2020–2024) in a natural population located in the Maolan National Natural Reserve, Libo county, Guizhou province, China (N 25°14′24′, E 108°03′29′, 520 m above sea level). Details on our methods and analyses are available as a Supplementary Appendix. Our findings support the hypothesis that these curious water-filled bracts in *Hemiboea* function by providing a favorable maternal environment for floral development under ecological conditions in which high temperatures, drought and calcium stress are likely to be pervasive.

When we artificially drained the fluids in the bracts by using a syringe every day after the bract began to swell (Appendix S1), the abortion rate of the flower buds was significantly higher than in unmanipulated intact bracts (Fig. 1H). There was no evidence that the differences in bud abortion between the two treatments was associated with damage to the bracts caused by the draining of the fluids. This is because, when we replaced the liquids in the bracts with purified water by using a syringe and sealed the holes by using Vaseline, the percentage of aborted flower buds was not significantly different from that of control flower buds (Fig. 1H).

To test the effect of fluids on the temperature inside the flower buds, we recorded the temperature in a natural population (Appendix S2). We found that, on sunny days, the temperature of the flower buds enclosed within the fluid-filled bracts was substantially lower than the ambient air temperature and lower than that of flower buds in which the fluids contained in the bracts were drained. However, these differences in temperature disappeared during night-time (Fig. 1I). Bracts filled with liquid lowered the temperature of flower buds by ≤3.6°C (Fig. 1I). Such a cooling effect likely results from the much higher specific heat of water than air. The flower-bud initiation stage is widely acknowledged as the most

temperature-sensitive period, particularly during the development of male gametophytes [10]. *Hemiboea magnibracteata* flowers from late July through to August, when temperatures tend to be highest in this subtropical region, with daytime maximum temperatures often exceeding 35°C [11,12]. Thus, it seems probable that the cooling effect of the womb-like bracts filled with fluid buffers floral development against high temperature stress in the subtropical karst environments.

Due to the structure of the epidermis of flower petals (e.g. higher epidermal surface conductance relative to neighboring leaves) [5], flowers commonly exhibit high rates of water loss, particularly associated with drought in hot and dry environments [13]. Under drought conditions, tender flower buds often dehydrate rapidly, even within a day without a new water supply [14]. Therefore, we infer that submerging flower buds in liquid during development may function to avoid desiccation in the stressful environments in which *H. magnibracteata* occurs.

Florivory can impact plant fitness and play an important role in driving the evolution of floral traits [15]. It is therefore possible that liquid in bracts might repel herbivores that consume flower buds [9,16]. However, during near continuous observation from 2021 to 2023, we found no evidence of herbivory on either intact bracts containing fluids or exposed flower buds in which bracts were removed. Moreover, we also found no evidence of herbivory on open flowers during flowering. These results cast doubt on the hypothesis that fluid-filled bracts function as a deterrent to floral herbivory.

Plants growing in karst regions have evolved a variety of mechanisms to adapt to the high levels of calcium in soils. For example, in some species, excess calcium is transferred from the cytoplasm and is sequestered in cell walls and stoma or in glands and epidermal hairs of leaves [11]. Our measurements of fluid in bracts indicated that they contained diverse elements, including Ca, Mg, N, P and K, with calcium having the highest concentration (77.3 ± 1.1 mg/L,), which was 1.7-fold higher than that in adjacent leaves (45.2 ± 0.8 mg/L; Student's *t*-test *P* < 0.001; Appendix S3). However, opposite patterns were observed for other elements (e.g. Mg, P and K; Appendix S3). While our experiment using a syringe and azaleine dye showed that the liquid in bracts is transferred to other plant parts, especially neighboring leaves, especially when plants are subjected to drought stress (Appendix S4), much of the fluid released during flowering occurred through bract dehiscence (Fig. 1E and F). Thus, our findings suggest that liquid-filled bracts may function as a protective calcium reservoir.

When the oval-like structures reached their maximum size, we subjected plants to drought stress by not watering them for 5 days (Appendix S4). Then, we injected azaleine into the bracts by using a syringe and the holes were then sealed by using Vaseline. We observed that some of the liquid was transferred to neighboring leaves within 2 hours, but not for well-watered plants (Appendix S4). Furthermore, dehydration-resistance experiments demonstrated that the water potential of leaves with neighboring bracts that were kept intact was significantly higher than that of leaves in which fluids in the neighboring bracts were drained (Fig. 1J and Appendix S4). Precipitation is abundant in subtropical karst regions; however, water scarcity is a persistent challenge for plants growing on limestone because of the limited water-retention capacity of the very shallow soil layer and high permeability of carbonate rocks [11]. Furthermore, although drought constrains performance at all growth stages, plants are more sensitive to water shortage during the flowering stage [17]. Therefore, we propose that the bracts of *H. magnibracteata* also serve as reservoirs of liquid that provide a vital water source for plant growth and reproduction. Thus, the bracts may serve several functions: they create a favorable microenvironment for the development of flower buds by protecting them against desiccation, they act as calcium reservoirs in high-calcium karst soils and they create a water reservoir for subsequent growth and reproduction through the ‘reuse’ of fluid in the bracts.

Prior to our study, there were no reports of terrestrial plants protecting flower buds in fluid-filled womb-like structures, although other protective mechanisms for protecting buds have been reported [9,16]. Our discovery raises the question of whether other taxa in Gesneriaceae possess the womb-like fluid-filled structures that we document here in *H. magnibracteata*. We surveyed other taxa of the family in China and, in addition to *Hemiboea*, we found an additional genus (*Raphiocarpus*) with similar structures. The minimally 28 species represent different lineages within a phylogeny that included 555 species of the estimated 922 Gesneriaceae species occurring in China (Appendix S5). The dispersed distribution of the womb-like structures across the phylogeny suggests that they likely evolved more than once in the family in this region.

In conclusion, our experiments are consistent with the hypothesis that the womb-like fluid-filled bracts of *H. magnibracteata* have several distinct functions related to the challenging ecological conditions of plant growth in the karst region of southwestern China. We have proposed that fluids in the bracts protect developing buds from excessive temperature and desiccation, but that they also may serve as a reservoir of calcium in the high-calcium karst soils as well as of water for growth under drought stress. Finally, we note that not all species of Gesneriaceae growing in sympatry with *H. magnibracteata* in the karst region possess fluid-filled bracts. This observation raises the question of how these species have adapted to this hot and drought-prone environment. Future comparative analyses in combination with detailed studies of physiological traits, environmental conditions and ecological genomics would be valuable to unveil the evolutionary origins of the curious case of ‘amniotic fluid’ in plants.

## Supplementary Material

nwaf195_Supplemental_Files
