# Supplementary material for: “Amniotic fluid” in womb-like flower bracts protects floral development and promotes drought resistance in karst habitats
Source: Natl Sci Rev. 2025 May 16;12(7):nwaf195. doi: 10.1093/nsr/nwaf195 (PMC12225171; doi:10.1093/nsr/nwaf195)
Supplement: nwaf195_Supplemental_Files [file nwaf195_supplemental_files.zip › Rrevised_Appendix_SI_for_resubmission.docx]

**Supporting Information for**

**“Amniotic fluid” in womb-like flower bracts protects floral development and promotes drought resistance in karst habitats**

**Yongpeng Ma^1, 2#^, Gang Yao^2#^, Yongquan Ren^3#^, Detuan Liu^1^, Yuanting Shen^2^, Wei Huang^4^, Yuewen Xu^2^, Spencer C.H. Barrett^5,^*, Hang Sun^4,^*, Bo Song^1,2,^***

^1^ Yunnan Key Laboratory for Integrative Conservation of Plant Species with Extremely Small Populations, Kunming Institute of Botany, Chinese Academy of Sciences, 132 Lanhei Road, Kunming 650201, China;

^2^ State Key Laboratory of Plant Diversity and Specialty Crops, Kunming Institute of Botany, Chinese Academy of Sciences, Kunming 650201, China;

^3^ College of Eco-Environmental Engineering, Guizhou Minzu University, Guiyang 550025, China;

^4^ Key Laboratory of Phytochemistry and Natural Medicines, Kunming Institute of Botany, Chinese Academy of Sciences, 132 Lanhei Road, Kunming 650201, China;

^5^ Department of Ecology and Evolutionary Biology, University of Toronto, 25 Willcocks Street, Toronto, ONM5S 3B2, Canada.

# These authors contributed equally

* Correspondence: songbo@mail.kib.ac.cn (B.S.); sunhang@mail.kib.ac.cn (H.S.); spencer.barrett@utoronto.ca (S.C.H.B)

**Appendix S1 Effect of fluid contained in womb-like bracts on the development of flower buds.**

We randomly selected 30 flowering plants and marked three oval-like bracts on each plant. Once these marked bracts began to swell, they were subjected to one of three treatments: (1) natural control: bracts left undisturbed; (2) drained: fluids in the bracts artificially drained using a syringe every day, and the holes were sealed using Vaseline; (3) replaced with water: fluids in the bracts extracted and replaced with **purified water using a syringe, and the holes were sealed with Vaseline to serve as a control for bract damage. We monitored flower development in these bracts daily, noting any instances of flower abortion, and calculated flower abortion rate as the ratio of the number of aborted flowers to the total number of flowers observed. To determine the impact of different treatments on flower development, we conducted a Kruskal-Wallis test, followed by a post hoc analysis using Mann-Whitney U test at 0.05 significance level.**

**Appendix S2** **Measurements of temperature variation in flower buds under three conditions: 1) immersed in fluid (control), 2) buds in bracts drained of fluid, and 3) ambient air adjacent to flower bracts.**

We recorded temperature inside the flower buds from plants growing naturally in the field in August using four-channel thermocouple data logger (Center 309; Center, Taiwan) equipped with four alloy needle-type sensor probes (1 mm in diameter and with an active tip length of 5 mm). To determine the effect of fluids on temperature inside the flower buds, we selected two swollen bracts on one plant. For one fluid-filled globe, we drained the fluids using a syringe and the other was treated as control. We recorded air temperature (*ca*. 60 cm above the ground, the height of flower stem) using an integrated thermistor (1,400-104 air temperature sensors; LI-COR, Lincoln, NE). Temperature was recorded at 1-min intervals and repeated three times.

**Appendix S3** **Measurements of components of fluids contained in the floral bracts.**

To determine the primary chemical components in fluids contained in bracts, we collected approximately 100 mL of fluid when bracts reached their peak size and the fluid was stored in 4 °C until testing. Additionally, approximately 100 mL of fluid was collected from adjacent leaves using the mechanical pressing method (1, 2), followed by centrifugation and filtration. We measured metallic elements using the PinAAcle 900T Atomic Absorption Spectrometer, whereas we analyzed nitrogen (N) and phosphorus (P) using the SEAL AA3 Continuous Flow Analyzer. All measurements were replicated three times.

**
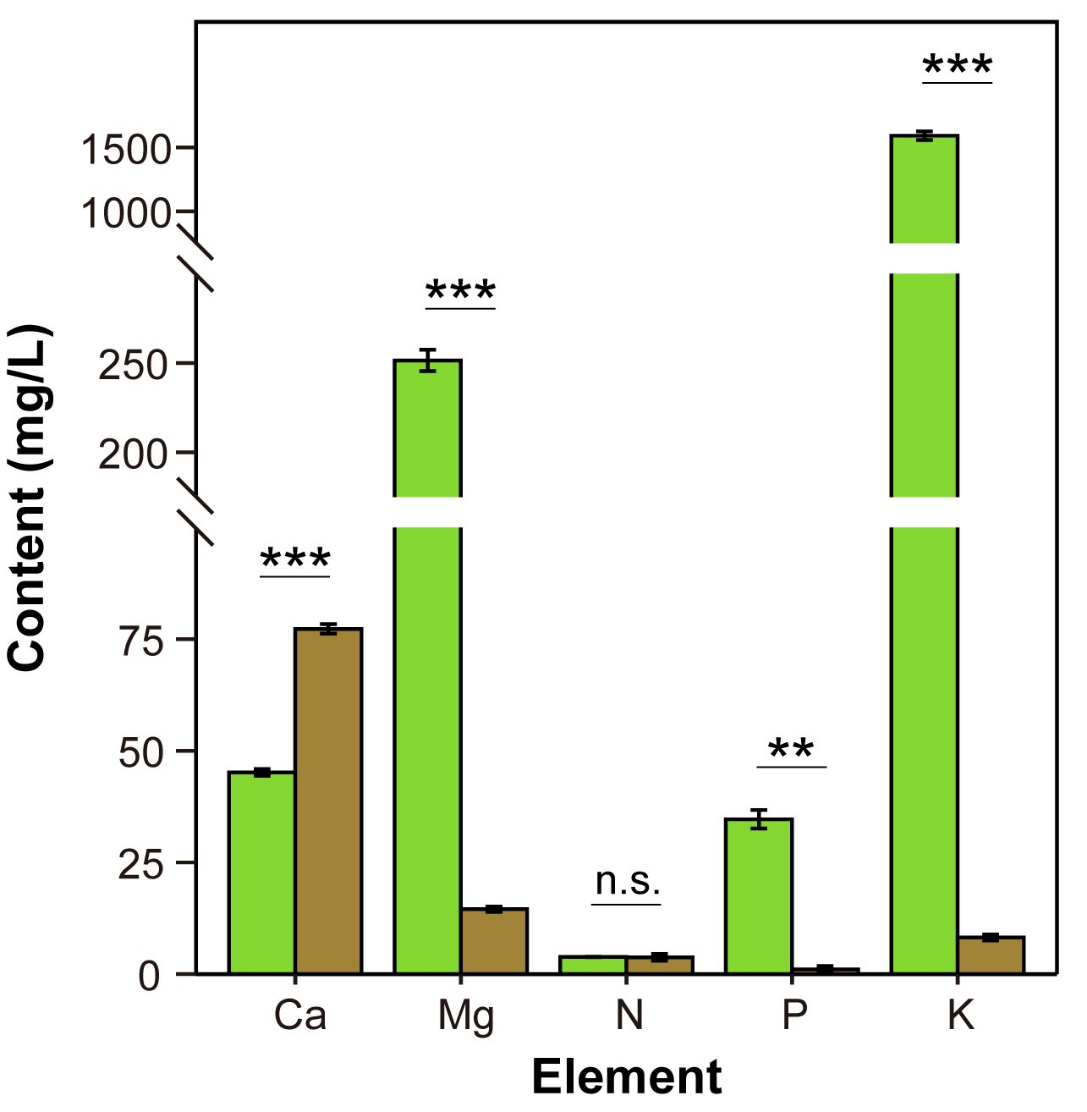
**

**Fig. S1** Contents (mean **± SE)** of various elements in the fluid from leaves (green) and bracts (brown) of ***Hemiboea magnibracteata* (*n* =3). Data are means ± SE. *** and ** indicate significant difference at *P* < 0.001 and 0.01, respectively.**

**References**

1. Mason, T.G., Phillis, E. (1939). Experiments on the extraction of sap from the vacuole of the leaf of the cotton plant and their bearing on the osmotic theory of water absorption by the cell. *Ann. Bot*. 3, 531-544.

2. Peasson, C.H. Rath, D.J. (2009). A hydraulic press for extracting fluids from plant tissue samples. *Ind. Crops Prod*. 29, 634-637.

**Appendix S4** **Fluids in bracts are transported to neighboring leaves and can enhance the tolerance of plants to drought.**

In May 2023, we collected ten ***Hemiboea magnibracteata*** plants from the field study site and planted them in pots (30 cm in diameter and 40 cm in height). These pots were placed in a glasshouse at Kunming Institute of Botany, Chinese Academy of Sciences, Kunming, Yunnan, Southwest China. When bracts reached their maximum size, plants were separated randomly into two groups. In the first, plants were not watered for five days; in the second, plants were watered every day. To visualize whether liquid in the bracts is transported to other plant parts, we injected azaleine into bracts using a syringe and holes were sealed using Vaseline (Fig. S2A, B). Subsequently, leaves were collected to prepare transverse sections to observe the microscopic characters under a stereoscope, especially the red pigment distribution in veins. We observed that fluids containing azaleine were transported to neighboring leaves 2 h later in plants subjected to drought stress (Fig. S2C, D), but not for well-watered plants (Fig. S2E, F).


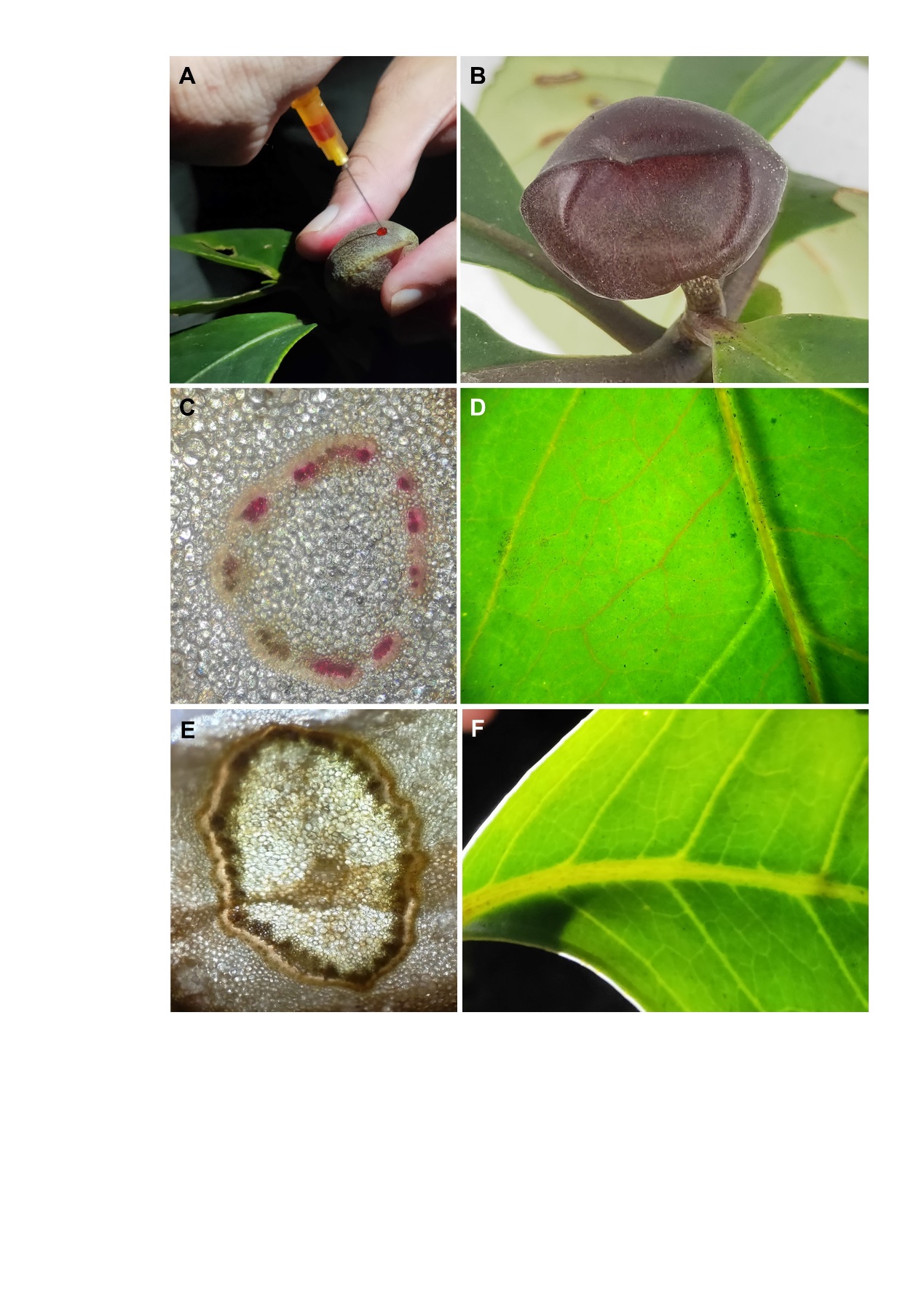


**Fig. S2** Injection of azaleine into the womb-like bracts of ***Hemiboea magnibracteata*** using a syringe and subsequent transportation of fluid injected with azaleine to neighboring leaves. A: azaleine is being injected into the womb-like bract using a syringe. B: A womb-like bract after azaleine injection. C, E: Cross sections of vein of neighboring leaves under a stereoscope: azaleine-infused red fluid is visible in the vein of leaves in drought-stressed plants (C), but absent in well-watered controls (E); D, F: Fluorescence microscopy of leaf veins: azaleine-infused red fluid is visible in drought-stressed plants (D), whereas no signal is detected in well-watered controls (F).

To determine the effect of fluids in bracts on the ability of plants to tolerate drought, we randomly selected six individuals with similar sized bracts. On each plant, one flower stem with one globe was retained. The plants were randomly separated into two groups. The fluid in bracts of the first group was drained using a syringe and holes were sealed using Vaseline, whereas the rest were left intact as a control. The plants were then removed from their pots to simulate the most extreme drought stress in the field. Immediately afterward, we measured leaf water potential for each plant using Model 1505D-EXP Pressure Chamber Instrument (PMS Instrument Company, USA). Five hours later, we measured the leaf water potential again. **To determine the impact of fluids in the bracts on leaf drought resistance, we conducted an independent-sample *t* test, at 0.05 significance level.**

**Appendix S5** **Phylogeny for Gesneriaceae taxa occurring in China with the maximum likelihood ancestral-state reconstruction of the womb-like structure.**

To infer the distribution of plants with womb-like structures in Gesneriaceae, we obtained a list of native plant species in this family from the Checklist of Plant Species in China (2024 Edition) (1). Each species was scored for the presence or absence of womb-like bracts based on published floras and trait databases, including efloras (<http://efloras.org/>), *Flora of China* (2), Plant Trait Database (TRY 2012), Gesneriaceae Resource Centre 2024 Checklist of *Hemiboea*. Species lacking detailed information were excluded.

The subfamilies and tribes of Gesneriaceae have been extensively studied through various targeted phylogenetic analyses, resulting in a wealth of available sequence data from Gesneriaceae taxa (3). We downloaded the nuclear ribosomal internal transcribed spacer (ITS) region sequences of the Gesneriaceae family from the Nucleotide database of the National Center for Biotechnology Information (NCBI). After cleaning, we obtained 945 samples from 555 species, out of the estimated 922 Gesneriaceae species occurring in China, with two species, *Syringa reticulate* and *Osmanthus austrocaledonicus* (Oleaceae), as outgroups. Precise multiple sequence alignment was performed using PRANK (v.170427) (4), following by trimming TrimAl (v1.4.rev15) (5). We constructed a phylogenetic tree for Gesneriaceae species in China using the Neighbor-Joining method (6) with the analysis performed in MEGA (7). We then mapped the presence or absence of womb-like structures onto the phylogenetic tree. Our results revealed that two genera, comprising 28 species and representing different lineages, exhibit womb-like structures within the phylogeny. The dispersed distribution of these womb-like structures suggests that they are likely to have evolved independently more than once within the family in this region.


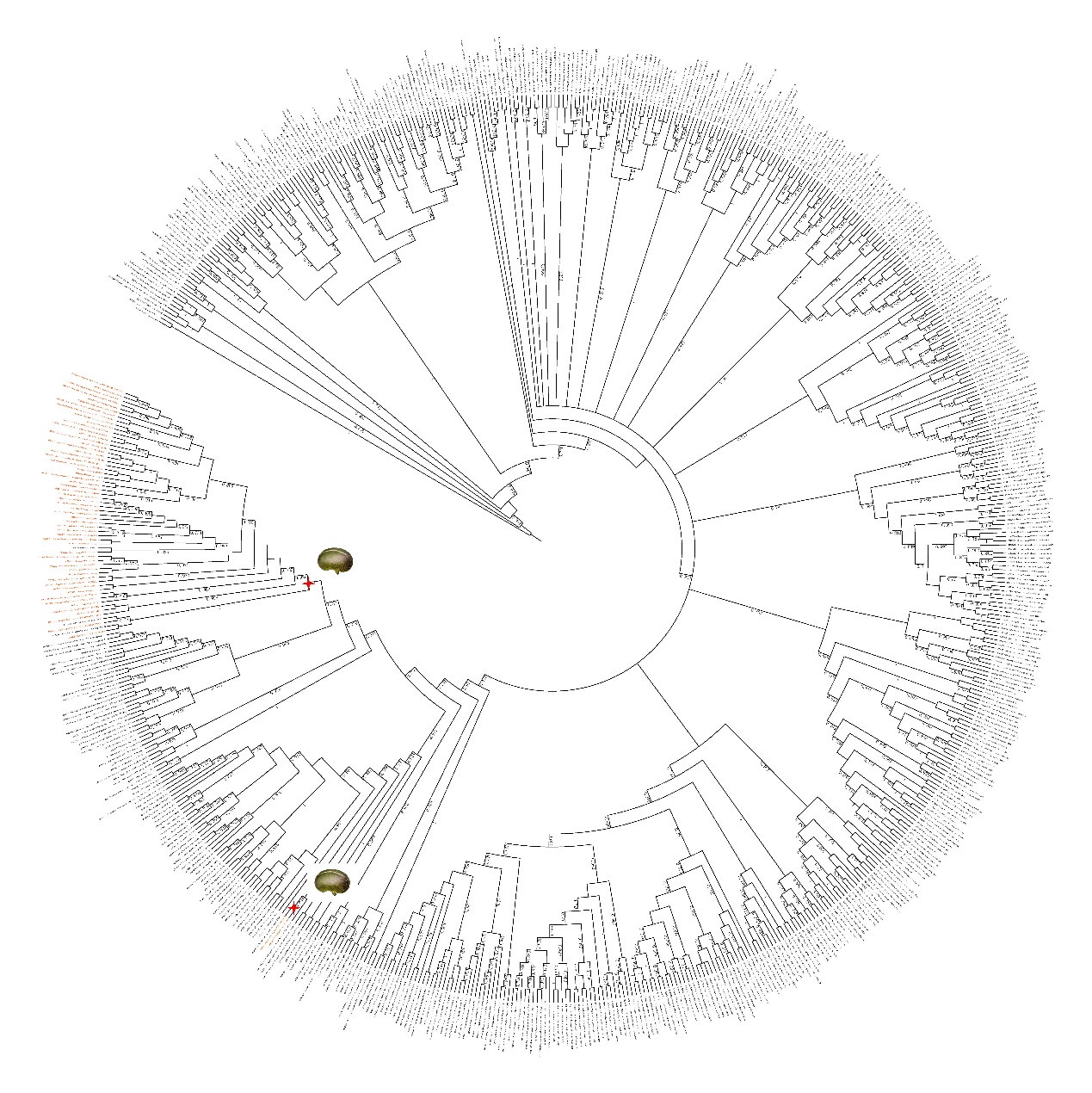


**Fig. S3 Neighbor-Joining (NJ) phylogenetic tree for** Gesneriaceae species occurring in China, **constructed using sequences from the** nuclear ribosomal internal transcribed spacer (ITS) region**. Species with** womb-like structures are marked in red. **Numbers at the nodes indicate NJ bootstrap support based on 1000 replicates.**

**References**

1. Checklist of plant species in China (2024 Edition). (2024). Plant Data Center of Chinese Academy of Sciences. CSTR: 34735.11.PLANTDATA.1476.

2. Z. Wu, P. Raven, Hong, D. Y. (1994-2013) Flora of China. (Beijing and St Louis:
Science Press and Missouri Botanical Garden Press). Online at efloras.org.

3. Roalson, E.H., and Roberts, W.R. (2016). Distinct processes drive diversification in different clades of Gesneriaceae. Syst. Biol. 65, 662–684. 10.1093/sysbio.syw012.4.

4. Loytynoja, A. & Goldman, N. (2008). Phylogeny-aware gap placement prevents errors in sequence alignment and evolutionary analysis. *Science* 320, 1632-1635.

5. Capella-Gutiérrez, S., Silla-Martínez, J.M., Gabaldón, T. (2009). trimAl: a tool for automated alignment trimming in large-scale phylogenetic analyses. *Bioinformatics* 25, 1972-1973.

6. Saitou, N. & Nei, M. (1987). The neighbor-joining method: a new method for reconstructing phylogenetic trees. *Mol. Biol. Evol*. 4, 406-425.

7. Hall, B.G. (2013). Building phylogenetic trees from molecular data with MEGA. *Mol. Biol. Evol*. 30, 1229-1235.
